# Cerebral hemorrhage during the active phase of SARS-CoV-2 infection in a patient with amyloid angiopathy: case report

**DOI:** 10.5935/0103-507X.20200098

**Published:** 2020

**Authors:** Amanda Ayako Minemura Ordinola, Samir Sari Osmar, Victor Hugo Rocha Marussi, Salomón Soriano Ordinola Rojas, Alex Machado Baeta, Feres Eduardo Chaddad Neto, Viviane Cordeiro Veiga

**Affiliations:** 1 BP - A Beneficência Portuguesa de São Paulo - São Paulo (SP), Brazil.; 2 Brazilian Research in Intensive Care Network (BRICnet) - São Paulo (SP), Brazil.

**Keywords:** Cerebral amyloid angiopathy, COVID-19, Coronavirus infections, SARS-CoV-2, Cerebral hemorrhage, Critical care, Intensive care units, Angiopatia amiloide cerebral, COVID-19, Infecções por coronavirus, SARS-CoV-2, Hemorragia cerebral, Cuidados críticos, Unidades de terapia intensiva

## Abstract

The neurological changes associated with COVID-19 have been frequently described, especially in cases of greater severity, and are related to multifactorial causes, such as endothelial dysfunction, inflammatory mediator release (cytokine storm), endothelial dysfunction and hypoxemia. We report the case of a female patient, 88 years old, with cerebral hemorrhage associated with amyloid angiopathy in the context of SARS-CoV-2 infection.

## Abstract (pt)

As alterações neurológicas associadas à COVID-19 têm sido frequentemente descritas, principalmente nos casos de maior severidade, e estão relacionadas a causas multifatoriais, como a disfunção endotelial, a liberação de mediadores inflamatórios (cytokine storm), a disfunção endotelial e a hipoxemia. Relatamos o caso de uma paciente do sexo feminino, 88 anos, com quadro de hemorragia cerebral associada à angiopatia amiloide, no contexto de infecção por SARS-CoV-2.

## INTRODUCTION

In March 2020, the World Health Organization (WHO) declared a pandemic caused by a new coronavirus, and currently, there are more than 10 million cases worldwide, of which 1.3 million are in Brazil.^(1)^ Increasing evidence has shown the neurotropism of SARS-CoV-2 and numerous related neurological manifestations.^(2,3)^

The mechanisms of neurovascular injury in COVID-19 are multifactorial, and vascular endothelial cell damage with consequent damage to the vasculature increases the risk of ischemic and hemorrhagic events.^(2)^

The objective of this study was to present a case report of a patient with cerebral amyloid angiopathy and hemorrhage during the active phase of SARS-CoV-2 infection.

## CASE REPORT

An 88-year-old female patient with a history of Alzheimer’s disease was hospitalized with worsening mental confusion for 4 days associated with periods of agitation, aggression and lack of appetite. She used sertraline and quetiapine continuously.

**Figure 1:**
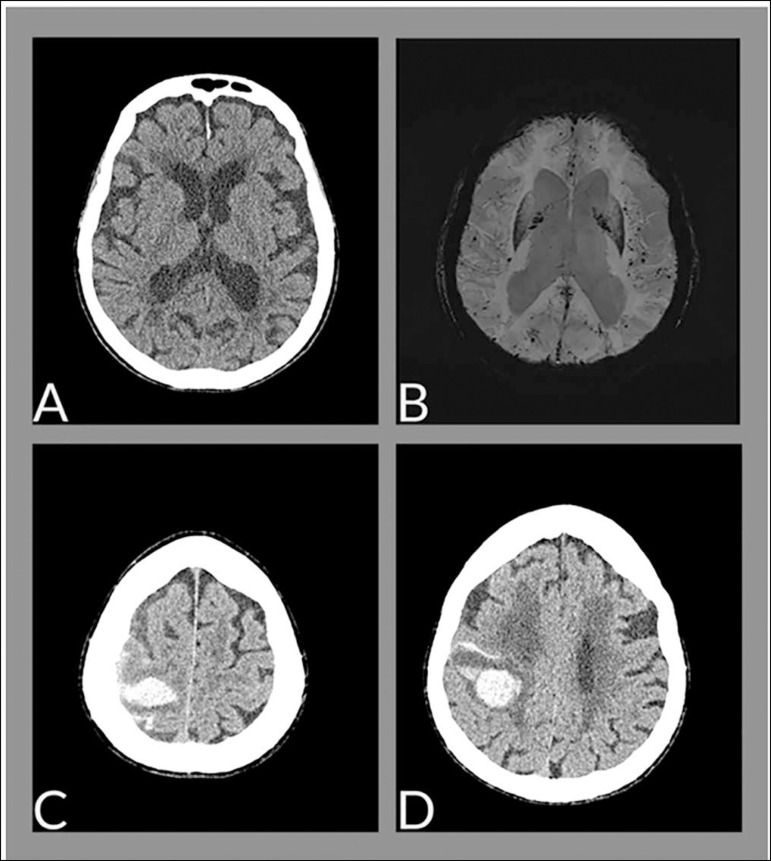
(A) Axial tomography image of the skull upon admission to the hospital, showing senile changes without evidence of hemorrhage. (B) Axial resonance image; SWI-minIP (susceptibility-weighted image) demonstrates multiple foci of hemosiderin deposition with a predominance in the periphery of the cerebral parenchyma (pattern suggestive of amyloid angiopathy). (C and D) Axial tomography images show intraparenchymal and subarachnoid hemorrhages in the right frontoparietal transition.

Upon admission, the patient presented with sleepiness and disorientation without focal deficits. Cranial tomography was performed, showing changes compatible with the patient’s age and no signs of acute changes (Figure 1A). A 6-hour electroencephalogram showed diffuse disorganization of brain electrical activity. A reverse transcription-polymerase chain reaction (RT-PCR) test for coronavirus 2019 disease (COVID-19) was requested upon hospital admission because of the ongoing pandemic and acute neurological disorder; the test was positive.

The patient remained under neurological surveillance, with adjustments to antipsychotic drugs and the introduction of pharmacological prophylaxis (enoxaparin) for venous thromboembolism. Magnetic resonance imaging was performed on the third day of hospitalization, showing the presence of multiple foci of hemosiderin deposition with a predominance in the periphery of the cerebral parenchyma (pattern suggestive of amyloid angiopathy) (Figure 1B).

On the fourth day of hospitalization, the patient presented with dysarthria and decreased motor strength in the left hemibody. Emergency brain tomography was performed, showing hemorrhage in the central and parieto-occipital sulcus on the right, in addition to the appearance of a recent intraparenchymal hemorrhage, measuring approximately 3.3 × 3.4 × 2.4cm (estimated volume of 14mL), affecting the precentral gyrus (Figures 1C and 1D).

For clinical support, the patient was admitted to a neurological intensive care unit, where heparin was suspended and neuroprotection measures were performed. After an evaluation, the neurosurgery team did not indicate surgical treatment. There were no changes in blood pressure or other vital signs. In the laboratory tests performed, there were no changes in the blood clotting test. However, there were changes in inflammatory markers (D-dimer, 1.411ng/mL; C-reactive protein (CRP), 3.15mg/dL, with a reference value lower than 1mg/dL). Ferritin and lactic dehydrogenase were within normal values.

The patient progressed with improvement in confusion, but motor deficits remained on the left side. On the 13th day, she presented involuntary facial movements, and a new electroencephalogram was requested, showing epileptiform activity of variable amplitude in both brain hemispheres, predominantly in the right anterior region. A new cranial tomography scan revealed greater regression of the subarachnoid hemorrhage content in the central and parieto-occipital sulcus as well as a greater reduction in the attenuation/partial metabolization of the recent intraparenchymal hematoma in the right perirolandic region, which mainly affected the postcentral gyrus, with an area of vasogenic edema more conspicuous in the neighboring cerebral parenchyma, similar to the mass effect characterized by the deletion of sulci between the local cortical gyri. Anticonvulsant drugs were started, and the patient’s condition improved without new crises (clinical or electrographic).

Currently, the patient remains in the rehabilitation process and still has motor deficits on the left side without other neurological changes.

## DISCUSSION

Mao et al.^(3)^ showed that 36.4% of hospitalized patients diagnosed with COVID-19 had neurological manifestations involving the central nervous system, peripheral nervous system, and skeletal muscle. In addition, patients with more severe disease were more likely to develop manifestations, and some had neurological changes as the initial manifestation of the disease.

A recent study with an English database showed neurological changes related to COVID-19 in 153 patients, including nine cases of cerebral hemorrhage. Ischemic strokes were more prevalent, with 57 cases. In this case series, 39 patients had an altered mental status.^(4)^

Therefore, patients admitted to emergency rooms with acute neurological conditions, in a pandemic context, should have SARS-CoV-2 as a differential diagnosis.

Divani et al., in a recent review on vascular events and COVID-19, showed a higher prevalence of ischemic events and, to date, few reports of cerebral hemorrhagic events in the literature.^(5)^

The main risk factors for primary intracranial hemorrhage include systemic arterial hypertension and amyloid angiopathy. For hemorrhage of secondary etiology, the factors include coagulopathy, rupture of arteriovenous malformations, venous thrombosis, rupture of aneurysms, tumors, hemorrhagic transformation, and vasculitis.^(6)^ The patient described in this report did not have hypertension, and brain MRI indicated amyloid angiopathy. The use of pharmacological prophylaxis for venous thromboembolism is also a factor that may be associated with bleeding.

The neurological changes associated with COVID-19 have multifactorial causes and are related to endothelial dysfunction, the release of inflammatory mediators (cytokine storm) and hypoxemia.^(2.7-9)^

In addition, COVID-19-related coagulopathy has been described; this condition is induced by an acute systemic inflammatory response in which there are increased blood coagulation markers, increased inflammatory markers and thrombocytopenia, which suggests a state of hypercoagulability.^(5)^ In our case, the patient presented changes in two inflammatory markers (CRP and D-dimer).

## CONCLUSION

Acute neurological changes in situations of COVID-19 are common, and given the current pandemic context, it is important to conduct an investigation in this group of patients to facilitate early diagnosis and management.
